# Unsupervised domain adaptation for the detection of cardiomegaly in cross-domain chest X-ray images

**DOI:** 10.3389/frai.2023.1056422

**Published:** 2023-02-09

**Authors:** Patrick Thiam, Ludwig Lausser, Christopher Kloth, Daniel Blaich, Andreas Liebold, Meinrad Beer, Hans A. Kestler

**Affiliations:** ^1^Institute of Medical Systems Biology, Ulm, Germany; ^2^Department of Diagnostic and Interventional Radiology, Ulm University Medical Center, Ulm, Germany; ^3^Department of Cardiothoracic and Vascular Surgery, Ulm University Medical Center, Ulm, Germany

**Keywords:** chest X-ray, cardiomegaly, deep learning, transfer learning, unsupervised domain adaptation

## Abstract

In recent years, several deep learning approaches have been successfully applied in the field of medical image analysis. More specifically, different deep neural network architectures have been proposed and assessed for the detection of various pathologies based on chest X-ray images. While the performed assessments have shown very promising results, most of them consist in training and evaluating the performance of the proposed approaches on a single data set. However, the generalization of such models is quite limited in a cross-domain setting, since a significant performance degradation can be observed when these models are evaluated on data sets stemming from different medical centers or recorded under different protocols. The performance degradation is mostly caused by the domain shift between the training set and the evaluation set. To alleviate this problem, different unsupervised domain adaptation approaches are proposed and evaluated in the current work, for the detection of cardiomegaly based on chest X-ray images, in a cross-domain setting. The proposed approaches generate domain invariant feature representations by adapting the parameters of a model optimized on a large set of labeled samples, to a set of unlabeled images stemming from a different data set. The performed evaluation points to the effectiveness of the proposed approaches, since the adapted models outperform optimized models which are directly applied to the evaluation sets without any form of domain adaptation.

## 1. Introduction

Although there have been several breakthroughs in the area of medical image analysis based on deep neural networks in the recent years, there are still a lot of issues to be dealt with in order for this specific type of technology to be effectively applied in a clinical setting. One of these issues is related to the generalization ability of trained deep neural networks on medical images stemming from different medical centers or recorded with different protocols. This is of particular interest in cases where the images share the same labels but have dissimilar appearances due to the different protocols used to record the data. The domain shift occurring in such settings causes a huge performance degradation of the optimized models, thus preventing a widespread adoption of such approaches in health care. Hence, a lot of efforts are being pulled in the specific area of domain adaptation (Ben-David et al., 2010) in order to alleviate this specific issue. The aim of domain adaptation is to effectively adapt a trained model to a data set whose data distribution is dissimilar to the one of the set used to optimize the model. Moreover, deep neural networks rely on a huge amount of annotated data in order to be effectively trained. However, such an annotation process is a very laborious task. Therefore, unsupervised domain adaptation approaches are proposed in order not only to adapt trained models to new data sets, but also in order to significantly reduce the cost of manual annotation needed for an effective optimization of an inference model. In the current work, unsupervised domain adaptation approaches are proposed and assessed for the detection of cardiomegaly in chest X-ray images. The objective of the conducted experiments is the adaptation of a deep neural network optimized on a huge set of labeled chest X-rays, on another set of unlabeled radiographs stemming from a different medical center. The classification task consists of the discrimination between posteroanterior chest radiographs of patients suffering from cardiomegaly and posteroanterior radiographs of healthy patients.

The remainder of the work is organized as follows. In Section 2, a summary of recent related works regarding the automatic detection of cardiomegaly, as well as unsupervised domain adaptation applied to medical image analysis is provided. A description of the proposed unsupervised domain adaptation approaches is subsequently provided in Section 3. The data used for the assessment of the proposed approaches as well as the corresponding experimental settings are described in Section 4. The results of the performed assessments are presented in Section 5, followed by a discussion of the presented results in Section 6. The work is subsequently concluded in Section 7 by summarizing the main findings of the conducted experiments and providing an outlook on potential future works.

## 2. Related works

Cardiomegaly refers to an abnormally enlarged heart, that can be caused by several factors and medical conditions such as high blood pressure, congenital heart disorders, coronary artery disease or pulmonary diseases, among others (Felker et al., 2000). In a clinical setting, one of the most commonly applied approach for the detection of cardiomegaly consists of measuring and interpreting the cardiothoracic ratio (CTR) (Danzer, 1919) of a posteroanterior (PA) chest radiograph. The CTR is defined as the ratio of heart to internal thoracic diameter, with a value higher than 0.50 usually pointing at a case of cardiomegaly, even though values up to 0.55 are considered by some radiologists as borderline (Pouraliakbar, 2018; Simkus et al., 2021). Despite its inherent simplicity, this specific approach still requires some manual computation of the CTR, followed by its interpretation in concordance with the corresponding chest radiograph by an expert. The resulting interpretation is therefore very subjective, while the entire process is time-consuming, and might also introduce discrepancies across different interpreters. Thus, in the last decades, several approaches in the domain of medical image processing involving Deep Neural Networks (DNNs) (LeCun et al., 2015), have been proposed in order to automatically perform the detection of instances of cardiomegaly based on chest radiographs. Such a system can assist clinicians in the diagnosis process by improving the efficiency of the interpretation process as well as significantly reducing the discrepancy among the resulting interpretations.

Two major categories of automatic cardiomegaly detection approaches emerge from the current literature: the first category consists of segmentation based approaches, which are characterized by the use of deep segmentation networks for the extraction of both thoracic and cardiac areas of interest. Subsequently, the CTR is computed based on the extracted areas of interest and a specific threshold is used to discriminate between normal instances and cardiomegaly instances. Que et al. (2018), propose a DNN architecture named CardioXNet for the detection of cardiomegaly from chest X-rays (CXRs). The architecture consists of a combination of two specific segmentation models (which are basically U-net DNNs Ronneberger et al., 2015) trained in an end-to-end manner to extract both cardiac and thoracic areas of the CXRs, respectively. The extracted regions of interest are subsequently used to compute the CTR and a threshold of 0.50 is used in order to distinguish between normal and cardiomegaly instances. A similar approach consisting of optimizing 2 distinctive segmentation models in order to extract both cardiac and thoracic areas of interest before computing the CTR is proposed by Lee et al. (2021).

The second category consists of classification based approaches, which do not involve any cardiac area or thoracic area segmentation process, nor a computation of the CTR. Such approaches rely on the optimization of specific feature representations stemming from the chest radiographs, for the discrimination between normal instances and cardiomegaly instances. Zhou et al. (2019) propose a transfer learning approach consisting of an ensemble of three DNNs (ResNet50 He et al., 2016, InceptionV3 Szegedy et al., 2016, Xception Chollet, 2017) pre-trained on the ImageNet data set (Krizhevsky et al., 2012). The features consisting of the output specific to the top fully connected layer of each single model are extracted and concatenated, before being fed into subsequent fully connected layers to generate the final output. Similarly, different authors (Bougias et al., 2020; Cardenas et al., 2020) evaluate several pre-trained DNNs for the detection of cardiomegaly based on transfer learning approaches, including such models as VGG16 and VGG19 (Simonyan and Zisserman, 2015), MobileNet (Howard et al., 2017), DenseNet121 (Huang et al., 2017), and EfficientNetB2 (Tan and Le, 2019). Bougias et al. (2020) replace the top fully connected layers of each pre-trained model by a logistic regression classifier before proceeding with the optimization of the resulting architecture. Meanwhile, Cardenas et al. (2020) replace the entire fully connected layers of each pre-trained model by a customized 3-layer multilayer perceptron (MLP). Uniquely the weights specific to the MLP are subsequently optimized during the training process. In contrast to the previous category of approaches, there is no segmentation involved and the performance of the proposed architecture is closely linked to the discrimination ability of the pre-trained models.

Sogancioglu et al. (2020), perform a comparison of both segmentation based cardiomegaly detection approaches and classification based detection approaches. The authors propose a segmentation based approach that relies on the optimization of U-net models for the extraction of both thoracic and cardiac regions of interest. Based on these areas of interest the CTR is computed and a threshold of 0.50 is applied to detect cases of cardiomegaly. The proposed classification approach relies on transfer learning to perform the inference task. Pre-trained models are fine-tuned and subsequently used to perform the classification task. The evaluation performed by the authors hints at segmentation approaches potentially outperforming classification approaches. Meanwhile, Grant et al. (2021) propose an innovative multi-modal approach consisting of simultaneously optimizing a deep neural network on both chest radiographs and non-imaging intensive care unit (ICU) data. These ICU data consist of vital sign values (e.g., heart rate, respiration rate), laboratory values (e.g., hemoglobin, glucose) and patient metadata (e.g., age, gender, ethnicity). The performed evaluation not only shows that non-imaging ICU data can be used to detect cardiomegaly instances at a certain extent, but also that the combination of non-imaging data with CXRs can improve the overall discrimination performance of an inference model.

These approaches have proven to be able to perform an automatic detection of cardiomegaly instances at a great extent. However, a significant performance degradation can be observed, when the optimized architectures are applied to data sets stemming from different clinical institutions or acquired with different protocols. This is mostly due to the domain shift observed in the new data sets. The assumption behind the ability of a machine learning inference model (DNNs in this specific case) to generalize to unseen samples is that both the training set and evaluation set are independent and identically distributed. However, depending on several factors such as dissimilar data recording procedures, the data distribution of the evaluation set can significantly differ from the data distribution of the training set, thus causing the observed performance degradation when evaluating the optimized model on the evaluation set. Hence, various domain adaptation (DA) approaches (Kouw and Loog, 2021) have been proposed in order to specifically deal with the domain shift between the training set and the evaluation set. In this setting, the training set is sampled from a specific source domain S and the evaluation set stems from a different but related target domain T. The goal of DA approaches is to optimize a model from the source domain in such a way that it generalizes in a target domain, by minimizing the difference between the data distribution of both domains. Kouw and Loog (2021) define three specific categories of DA approaches: first, *sample-based approaches* which consist of weighting individual samples from the source domain during the optimization process of a model based on the relevance of these samples for the target domain. Such approaches as data importance-weighting (Cortes and Mohri, 2014) or class importance-weighting (Lipton et al., 2018) belong to this category; second, *feature-based approaches* which consist of optimizing domain invariant feature representations in such a way that a model trained in the source domain can easily be applied to the target domain without any significant performance degradation. This category encompasses such approaches as the Domain-Adversarial Neural Network (DANN) (Ganin et al., 2016) or the deep reconstruction-classification network (DRCN) (Ghifary et al., 2016); third, *inference-based approaches* which incorporate the adaptation procedure into the parameter optimization process through the use of specific constraints during the optimization procedure. Such approaches as Cycle Self-Training (CST) (Liu et al., 2021) or Minimax Entropy (MME) (Saito et al., 2019) belong to this category.

Domain adaptation approaches have been developed and applied to the analysis of chest X-ray images in a cross-domain setting such as in the work of Tang et al. (2019), where the authors propose a task-oriented unsupervised adversarial network (TUNA-Net) for pneumonia recognition in cross-domain chest X-ray images, which is basically a cycle-consistent generative adversarial network. Zhang et al. (2022) propose an unsupervised domain adaptation approach for the cross-domain classification of thorax diseases, characterized by the application of three specific types of constraints [Domain-Invariance (DI), Instance-Invariance (II), and Pertubation-Invariance (PI)] for the optimization of domain invariant feature representations. Meanwhile, Pham et al. (2021) propose an unsupervised adversarial domain adaptation method for multi-label classification tasks. We refer the reader to the works presented by Çalli et al. (2021) and Guan and Liu (2022) for further insights into deep learning approaches as well as DA approaches applied in the area of medical image analysis.

In the current work, unsupervised domain adaptation approaches are proposed and evaluated in a cross-domain setting for the detection of cardiomegaly instances based on posteroanterior chest radiographs. The described approaches aim to improve the generalization ability of specific deep neural networks by performing the models' optimization on a large set of labeled samples (Source Domain S), and adapting the optimized models to another set of samples (Target Domain T) stemming from a different medical center. The source domain consists of the publicly available PadChest data set (Bustos et al., 2020), which is a large set of labeled CXR images stemming from the San Juan Hospital in Spain. The evaluation of the approaches is performed on two different target domains: first a significantly smaller custom set of chest radiographs collected at the department of diagnostic and interventional radiology of the Ulm University Medical Center in Germany, and second, a publicly available OpenI (Demner-Fushman et al., 2012) data set (Demner-Fushman et al., 2016) consisting of CXR images collected from various hospitals of the Indiana School of Medicine in the United States of America. Since the size of the OpenI data set is significantly higher as the size of the set stemming from the Ulm University Medical Center, similar evaluation experiments are performed using the OpenI data set as the source domain.

## 3. Methodology

In the current settings, a data set X of size *n* stemming from the source domain S, with its corresponding set of labels Y, is given. Likewise, an unlabeled data set Z of size *m*, stemming from the target domain is also given. However, both source and target domains share an identical label space. The goal of the proposed unsupervised domain adaptation approaches is to improve the generalization ability of an inference model $f_{S}:X\to Y$ trained on the source domain, on the target domain by utilizing both labeled samples drawn from the source domain and unlabeled samples drawn from the target domain.

**Figure 1:**
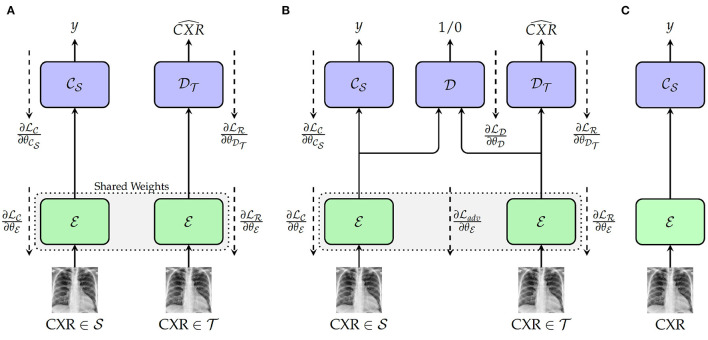
Proposed approaches. **(A)** Training with deep reconstruction domain adaptation (DRDA): E: Encoder (a single encoder is used to extract the features from both source and target domains' samples); $\theta_{E}$: weights of the encoder; $C_{S}$: Source domain classifier; $\theta_{C_{S}}$: weights of the classifier; $D_{T}$: Target Domain Decoder; $\theta_{D_{T}}$: weights of the decoder. **(B)** Training with regularized deep reconstruction domain adaptation (RDRDA): E: Encoder (a single encoder is used to extract the features from both source and target domains' samples); $C_{S}$: Source domain classifier; $D_{T}$: Target Domain Decoder; D: Domain Discriminator; $\theta_{D}$: weights of the domain discriminator. **(C)** Testing: during this phase, both optimized encoder and source domain classifier are used to perform the classification of unseen samples from the target domain.

The first approach depicted in Figure 1A consists of a Deep Reconstruction Domain Adaptation (DRDA) approach and is inspired by the work presented in Ghifary et al. (2016). A shared encoder E extracts features from samples drawn from both source and target domains. The extracted features from the labeled samples of the source domain are subsequently fed into a classification model $C_{S}$ to generate the corresponding labels. Concurrently, the features extracted from the unlabeled samples of the target domain are fed into a decoder $D_{T}$ in order to reconstruct the corresponding CXR. During back-propagation, the classifier's weights $\theta_{C_{S}}$ are updated based on the corresponding classification loss function $L_{C}$, while the encoder's weights $\theta_{D_{T}}$ are updated using a corresponding reconstruction loss $L_{T}$. The encoder's weights are updated with the following loss function:


(1) $$\begin{array}{l} L=\lambda_{c}L_{C}+\lambda_{r}L_{R} \end{array}$$

with λ_*c*_+λ_*r*_ = 1. By jointly optimizing the encoder, as well as the source domain classifier and the target domain decoder, a feature representation is generated which is able to perform the discrimination between the classes of the source domain, while encoding useful features from the target domain. Therefore, both optimized encoder and source domain classifier can subsequently be combined as a model and evaluated on unlabeled samples drawn from the target domain (see Figure 1C).

The DRDA approach is subsequently extended to a Regularized Deep Reconstruction Domain Adaptation (RDRDA) approach by adding an adversarial loss characterized by a Domain Discriminator model D as depicted in Figure 1B. The regularization performed through the additional adversarial loss adds an additional constraint to the features generated by the encoder. The extracted features should not only be optimized in order to perform both the classification of samples from the source domain as well as the reconstruction of the samples from the target domain, but also the discriminator should not be able to distinguish between the features of the samples from either domains, thus reinforcing the domain invariance characteristic of the extracted features. The domain discriminator model takes as input the features extracted from both source and target domains' samples and performs a discrimination between both domains (the samples from the source domain are labeled as 1, while the samples of the target domain are labeled as 0). Its weights $\theta_{D}$, are optimized using the corresponding classification loss function $L_{D}$. An adversarial loss $L_{adv}$ is subsequently used additionally in order to perform the optimization of the encoder's weights, with the corresponding final loss formulated as follows:


(2) $$\begin{array}{l} L=\lambda_{c}L_{C}+\lambda_{r}L_{R}+\lambda_{d}L_{adv} \end{array}$$

with λ_*c*_+λ_*r*_+λ_*d*_ = 1. Following the optimization of the models, the resulting domain invariant feature representations can be used in order to perform the classification task in the target domain.

In the current work, the source domain classification loss $L_{C}$ is a categorical cross-entropy loss, while the domain discrimination loss $L_{D}$ is a binary cross-entropy loss. Furthermore, the target domain reconstruction loss $L_{R}$ is basically a mean-squared-error loss:


$$\begin{array}{lll} L_{C} & = & \frac{1}{bs}\overset{bs}{\underset{k=1}{\sum }}\left(w_{k}\left(-\overset{c}{\underset{j=1}{\sum }}y_{k,j}log\left({ŷ}_{k,j}\right)\right)\right), \end{array}$$

(3) $$\begin{array}{l} \text{with}{ŷ}_{k}=\left({ŷ}_{k,1},\ldots ,{ŷ}_{k,j},\ldots ,{ŷ}_{k,c}\right)=C_{S}\left(E\left(x_{k}\right)\right) \end{array}$$

(4) $$\begin{array}{l} L_{D}=-\frac{1}{bs}\left(\overset{bs}{\underset{k=1}{\sum }}log\left(D\left(E\left(x_{k}\right)\right)\right)+\overset{bs}{\underset{k=1}{\sum }}log\left(1-D\left(E\left(z_{k}\right)\right)\right)\right) \end{array}$$

(5) $$\begin{array}{l} L_{R}=\frac{1}{bs}\overset{bs}{\underset{k=1}{\sum }}z_{k}-D_{T}\left(E\left(z_{k}\right)\right){}_{2}^{2} \end{array}$$

with $y_{k}=\left(y_{k,1},\ldots ,y_{k,j},\ldots ,y_{k,c}\right)\in Y$ (*c* being the number of classes), $x_{k}\in X$, *w*_*k*_ corresponding to the weight of the sample *x*_*k*_ (in order to deal with data imbalance), $z_{k}\in Z,\forall k$, and *bs*∈ℕ_>0_ standing for batch size. The adversarial loss is basically an inverted label loss as proposed in Tzeng et al. (2017) and is defined as follows:


(6) $$\begin{array}{l} L_{adv}=-\frac{1}{bs}\overset{bs}{\underset{k=1}{\sum }}log\left(D\left(E\left(z_{k}\right)\right)\right). \end{array}$$

By optimizing the weights of the encoder based on the adversarial loss, the discriminator is unable to distinguish between samples stemming from either source or target domains, thus the encoder generates domain invariant feature representations.

## 4. Materials and experimental settings

In the following section, a description of the data sets used for the evaluation of the proposed approaches is provided. The data pre-processing steps applied to the CXRs are subsequently described, followed by a description of the experimental settings.

###  4.1. Chest X-rays data sets

The evaluation of the domain adaptation approaches is performed on three CXR data sets. The largest one consists of the publicly available *Pathology Detection in Chest radiographs* (PadChest) data set (Bustos et al., 2020). It consists of 160, 868 CXRs from 69, 882 patients, recorded at the San Juan Hospital in Spain between 2009 and 2017. The CXRs were recorded in six different positions, including standing posteroanterior (PA) and lateral (L) views, anteroposterior (AP) supine and erect views, lordotic and oblique sternum views. Around 27% of the entire data set was manually annotated by trained physicians into a total of around 170 distinct categories of radiographic findings, including cardiomegaly. Cases where no anomalies were found were subsequently annotated as normal CXRs. The remaining 73% of the data set was automatically annotated using an attention-based recurrent neural network (trained using the set of manually annotated CXRs). In the current work, experiments are performed based uniquely on manually annotated CXRs recorded in a standing posteroanterior view. Furthermore, models are optimized on a training set consisting of uniquely two categories of CXRs, namely cardiomegaly and normal, since the focus of the current work is on the detection of instances of cardiomegaly. A stratified random sampling is applied on the resulting data set (manually labeled PA CXRs of cardiomegaly and normal instances) in order to generate the training, validation and testing sets used in the subsequent experiments to perform the evaluation of the proposed approaches. Around 10% of the entire data set is used as testing set, 10% of the entire data set is used as validation set, and the remaining 80% is used as training set. The resulting data distribution is depicted in Table 1.

**Table 1:** Data distribution.

	**CXR PadChest**			**CXR OpenI**			**CXR Ulm**		
	**Training set**	**Validation set**	**Testing set**	**Training set**	**Validation set**	**Testing set**	**Training set**	**Validation set**	**Testing set**
Cardiomegaly	1, 712	214	214	167	83	84	39	20	20
Normal	6, 966	871	871	697	350	349	26	13	13
Total	8, 678	1, 085	1, 085	864	433	433	65	33	33

Following some stratified random sampling of the datasets, the data distribution of both cardiomegaly and normal classes for each dataset is depicted.

The second data set consists of the publicly available *Indiana University chest X-ray Collection* (Demner-Fushman et al., 2016). It consists of around 7, 470 manually labeled CXRs, recorded in both lateral and PA views and stemming from various hospitals of the Indiana University School of Medicine, in the USA. The data set is extracted from the National Library of Medicine (NLM) using the Open Access Biomedical Search Engine (OpenI) (Demner-Fushman et al., 2012). OpenI constitutes a multi-modal web-based data retrieval system that enables the search and retrieval of medical images (such as magnetic resonance imaging (MRI), X-ray images, computed tomography (CT) scans, ultrasound images) using a combination of textual and visual queries. As mentioned earlier, the current work focuses on the detection of cardiomegaly based on PA CXRs. Thus, the data retrieved for the evaluation of the proposed approaches consist of PA CXRs labeled as cardiomegaly or normal. Following a stratified random sampling of the retrieved data, around 25% of the data is used as testing material, while 25% of the data is used as validation set. The training set consists of the remaining 50% (see Table 1 for the corresponding data distribution).

The third data set consists of manually annotated PA CXRs from a total of 131 patients (31 female and 100 male), collected within a study at the Department of Diagnostic and Interventional Radiology of the Ulm University Medical Center, in Germany. The study was compliant with regards to the health insurance portability and accountability act (HIPAA). The ethics board of the Medical Faculty and the University Hospital approved this retrospective data evaluation study and waived the informed consent requirement (No. 115/21). Two radiologists verified and relabeled the data set. Images were labeled as cases of cardiomegaly based on a CTR threshold of 0.55. A stratified random sampling of the data set in training (50% of the entire data set), validation (25% of the data set) and testing (25% of the data set) sets is performed and subsequently used for the evaluation of the proposed approaches. The data distribution of the resulting sets is depicted in Table 1.

###  4.2. Data pre-processing

**Figure 2:**
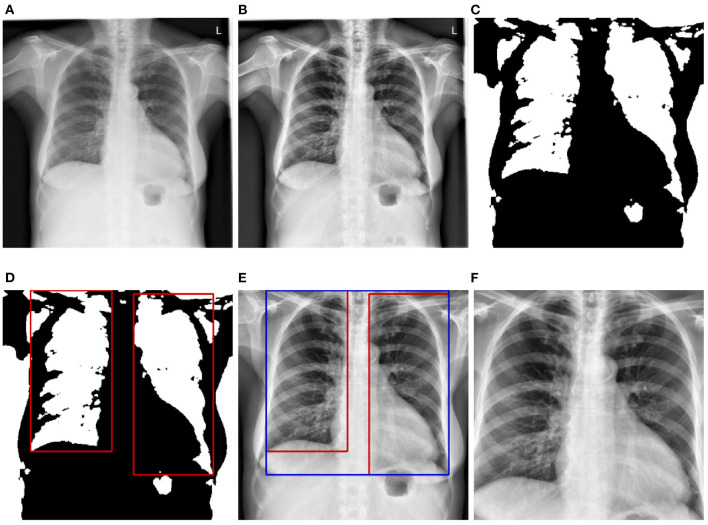
CXR pre-processing steps. **(A)** Grayscale CXR. **(B)** Histogram equalized CXR. **(C)** Binarized CXR. **(D)** Lungs localization. **(E)** Area of interest localization. **(F)** Cropped area of interest (pre-processed CXR).

Before being fed into the designed neural networks, CXRs have to be pre-processed in order to significantly reduce the amount of noise within the images, which hinders an optimal optimization of the parameters of the neural networks, resulting into overall sub-optimal classification performances. Moreover, since the data used for the evaluation of the trained deep neural networks stem from another domain, the pre-processing steps also help homogenizing the structure of the data at a certain extent (since the data stemming from both source and target domains go through the exact same pre-processing steps). In the current work, the pre-processing consists of localizing and extracting the chest cavity from each CXR and subsequently using this specific area to perform the evaluation of the proposed approaches. First, CXRs are converted into gray-scale images (see Figure 2A). Secondly, histogram equalization is applied on the resulting images in order to enhance the images' contrasts by applying the contrastive limited adaptive equalization (CLAHE) approach (Zuiderveld, 1994) (see Figure 2B). The resulting images are filtered using a 3 × 3 Gaussian filter. Further noisy details appearing at the edges of the CXRs are also filtered out by zooming into the images with a range set as follows [0.1, 0.1]. Furthermore, a binarization of the filtered images is performed, followed by the application of a set of morphological transformations (erosion and dilation) using a 3 × 3 kernel in order to generate masks over the lungs in the CXRs (see Figure 2C). The contours of the masks are subsequently computed and bounding rectangles around the resulting contours are generated (see Figure 2D). The extreme points of the bounding rectangles are subsequently used to generate a bounding rectangle identifying the chest cavity (see Figure 2E). This specific area of interest is subsequently cropped out of each cxr (see Figure 2F), resized to the shape 299 × 299 × 3 (the number of channels is obtained through the duplication of the cropped CXR by the corresponding amount), and constitutes the input for the designed deep neural networks.

###  4.3. Experimental settings

In order to perform the assessment of the proposed unsupervised domain adaptation approaches, several experiments are conducted. A baseline of the classification performance specific to each data set is computed by performing a transfer learning classification experiment using uniquely the labeled training set specific to each data set and evaluating the trained model on the corresponding testing set (T→T). Furthermore, in order to assess the performance improvement achieved by applying the domain adaptation approaches, an additional transfer learning classification experiment is performed by training a model uniquely on a source data set (using uniquely the corresponding training set) and evaluating the trained model on the target data set (the evaluation is performed on the corresponding testing set) (S→T). This is basically a cross-domain classification experiment without any domain adaptation involved, with the source domain being the labeled training set of a specific data set and the target domain being the testing set of a different data set. Finally, unsupervised domain adaptation experiments are conducted by adapting each model using both the labeled training set of the source domain and the unlabeled training set of the target domain. The adapted model is subsequently evaluated on the testing set of the target domain. Since the goal of the current work is to adapt a model trained on a set of labeled samples to some set of samples stemming from a different domain, the larger sets are used as source domains in order for an effective classification model to be optimized using a large number of labeled samples. The Ulm data set being particularly small as it can seen in Table 1, we perform the experiments once using the OpenI CXR data set as source domain and the Ulm CXR data set as target domain, and twice using the PadChest data set as source domain, with both OpenI CXR and Ulm CXR data sets as target domains subsequently. The architectures of the neural networks designed for the performed experiments are described as follows:

#### 4.3.1. Encoder (E)

**Figure 3:**
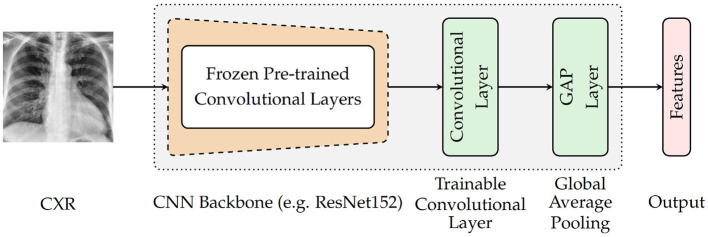
Encoder's architecture.

As depicted in Figure 3, the architecture specific to the encoder involves a backbone consisting of pre-trained convolutional layers, followed by an additional and single trainable convolutional layer and a subsequent global average pooling (GAP) layer. The backbone is generated by removing the top fully connected (FC) layers of a pre-trained deep neural network and freezing the remaining convolutional layers (which means that the weights of the corresponding convolutional layers are not modified during the optimization process). The encoder takes as input a CXR and generates as output a feature vector that is subsequently fed into the following module of the classification system. For the current experiments, four different CNN models trained on the ImageNet database (Russakovsky et al., 2015) are evaluated as pre-trained base models (CNN Backbone): ResNet152 (He et al., 2016), DenseNet121 (Huang et al., 2017), Xception (Chollet, 2017), and InceptionV3 (Szegedy et al., 2016). The subsequent trainable layer performs a convolution operation using a total of 1, 024 filters consisting of 3 × 3 kernels and 1 × 1 strides, followed by a batch normalization layer and a subsequent Rectified Linear Unit (ReLU) activation. Following the subsequent global average pooling, a feature vector of size 1, 024 is generated.

#### 4.3.2. Target domain decoder ($D_{T}$)

The target decoder takes as input the feature vector generated by the encoder and reconstructs the corresponding target CXR. The architecture consists of a series of transposed convolutional layers, each followed by a ReLU activation except for the last layer where a linear activation function is applied.

#### 4.3.3. Source domain classifier ($C_{S}$)

The source classifier takes as input the features specific to the samples from the source domain and generated by the encoder. The generated output consists of the class prediction of the corresponding CXR (cardiomegaly or normal). The architecture of the source classifier consists of two dense layers (fully connected layers). The first layer uses a ReLU activation function and the second layer uses a Softmax activation function.

#### 4.3.4. Domain discriminator (D)

The domain discriminator takes as input the features of the samples from both domains and performs a discrimination between the target domain and source domain (samples from the source domain are labeled as 1 while samples from the target domain are labeled as 0). Its architecture also consists of two dense layers. The first layer uses a ReLU activation function and the second layer uses a Sigmoid activation function. The details of the architectures can be found in Table 2.

**Table 2:** Neural networks' architectures.

**Layers**	**No. Filters/** **units**	**Kernels**	**Strides**	**Padding**
**Target domain decoder (D**_*T*_ **)**				
Dense	8 × 8 × 128	−	−	−
Reshape	−	−	−	−
2 × Conv2DTranspose	128	(3 × 3)	(2 × 2)	−
1 × Conv2DTranspose	64	(3 × 3)	(1 × 1)	−
1 × Conv2DTranspose	64	(3 × 3)	(2 × 2)	same
1 × Conv2DTranspose	64	(3 × 3)	(2 × 2)	−
1 × Conv2DTranspose	32	(3 × 3)	(2 × 2)	−
1 × Conv2DTranspose	32	(3 × 3)	(1 × 1)	same
1 × Conv2DTranspose	3	(3 × 3)	(1 × 1)	same
**Source domain classifier** ($C_{S}$)				
Dropout	−	−	−	−
Dense	512	−	−	−
Dropout	−	−	−	−
Dense	2	−	−	−
**Domain discriminator (** D **)**				
Dropout	−	−	−	−
Dense	512	−	−	−
Dropout	−	−	−	−
Dense	1	−	−	−

The dropout rate is set empirically to 0.25.

All architectures are trained using the Adaptive Moment Estimation optimization algorithm (Adam) (Kingma and Ba, 2015) with a fixed learning rate set to 10^−6^, for a total of 200 epochs. Due to memory constraints, each architecture is also trained with a fixed batch size of 16. During the domain adaptation experiments, since the size of the labeled source data set is different from the one of the unlabeled target data set, batch training is implemented in such a way that at each single epoch, CXR images are evenly sampled from each data set (source and target). The same amount of samples (the specified batch size *bs* = 16 in the Equations 3–6) is randomly selected from each data set and fed into the architecture to optimize its parameters. The next training epoch begins, once each sample of the training set specific to the source domain has been used at least once for the optimization of the parameters of the deep neural network, during a single epoch. In the case of the deep reconstruction domain adaptation (DRDA) approach, the weighting parameters defined in Equation (1) are set empirically as follows: λ_*c*_ = λ_*r*_ = 0.5. In the case of the regularized deep reconstruction domain adaptation (RDRDA), the weighting parameters defined in Equation (2) are set empirically as follows: λ_*c*_ = λ_*r*_ = 0.45;λ_*d*_ = 0.1. The implementation and the evaluation of the proposed approaches are performed with the libraries Tensorflow (Abadi et al., 2016), Keras (Chollet, 2015), and Scikit-learn (Pedregosa et al., 2011).

In order to account for the imbalanced data distribution within each single labeled training set, samples are weighted as follows. Given a set of samples X, with the corresponding set of labels Y such that ∥X∥=n and $\forall y_{k}\in Y,y_{k}\in \left\{\left(1,0\right),\left(0,1\right)\right\}$: we define $X^{-}=\left\{x_{k}\in X|y_{k}=\left(1,0\right)\right\}$ ($n^{-}=∥X^{-}∥$) and $X^{+}=\left\{x_{k}\in X|y_{k}=\left(0,1\right)\right\}$ ($n^{+}=∥X^{+}∥$):


(7) $$\forall x_{k}\in X,\;w_{k}=\{\begin{array}{ll} \frac{n^{-}}{n} & \text{if }x_{k}\in X^{+}\\ \frac{n^{+}}{n} & \text{otherwise} \end{array}$$

where *w*_*k*_ is the weight specific to the sample *x*_*k*_ and *n* = *n*^−^+*n*^+^. The performance metrics used to conduct the assessment of the proposed approaches are defined in Table 3. In the current work, the positive class consists of samples labeled as cases of cardiomegaly, while the negative class consists of samples labeled as normal.

**Table 3:** Evaluation metrics.

**Sensitivity (Sens)**	** $\frac{TP}{TP+FN}$ **
Specificity (Spec)	$\frac{TN}{TN+FP}$
Geometric Mean (G-Mean)	$\sqrt{Sensitivity\times Specificity}$
Accuracy (Acc)	$\frac{TP+TN}{TP+TN+FP+FN}$
Precision (Prec)	$\frac{TP}{TP+FP}$
F1-Score (F1)	$\frac{2\times Sensitivity\times Precision}{Sensitivity+Precision}$

TP, True positive (correctly classified samples of the positive class); FP, False positive (incorrectly classified samples of the negative class); TN, True negative (correctly classified samples of the negative class); FN, False negative (incorrectly classified samples of the positive class).

## 5. Results

As described in Table 1, each data set is characteristically skewed, with the cardiomegaly class being the minority class for both the PadChest and OpenI data sets, and with similar imbalance ratios: cardiomegaly instances account for $\frac{1712}{8678}\approx 19.73\%$ of the training set in the case of the PadChest data set, while in the case of the OpenI data set, a similar ratio of $\frac{167}{864}\approx 19.34\%$ can be observed. Concerning the Ulm data set, normal instances constitute the minority class with an imbalance ratio of $\frac{26}{65}\approx 40\%$. Since the goal is to optimize a model that performs at a satisfactory extent on both majority and minority classes, the most relevant metrics used to assess the performance of the proposed approaches consist of the Sensitivity (true positive rate), Specificity (true negative rate) and the Geometric Mean of Sensitivity and Specificity (see Table 3). The overall classification accuracy in the case of imbalanced data sets is unreliable on its own, since it is biased toward the majority class. Hence, relying on the classification accuracy alone in order to assess the performance of the proposed approaches would result in misleading interpretations of the achieved performances.

The impact of the cropping of the specified area of interest during the pre-processing of the CXR images (see Section 4.2) can be seen in Table 4. The depicted results stem from a classification task, consisting of optimizing a model using both training and validation sets specific to the PadChest data set. The optimized model is subsequently applied on the testing set specific to the PadChest data set. Both optimization and inference tasks are performed twice for each pre-trained model: firstly with the histogram equalized images without the cropping step, and secondly with the histogram equalized and cropped CXR images. While the results are similar in both cases, there is a slight but systematic performance improvement across all architectures in terms of G-Mean and area under the receiver operating characteristic curve (AUC) when the cropping procedure is additionally applied. These results point at the fact that noisy information have effectively been removed from the images. Thus, the proposed pre-processing steps are beneficial for the discrimination between normal cases and cases of cardiomegaly. However, in cases where the classification task consists of different types of pathologies or lung diseases, caution should be applied while defining a specific area of interest and subsequently cropping the CXR images, in order to avoid removing potentially useful information from the images before performing the inference task. The remaining experiments are performed on the histogram equalized and cropped CXR images.

**Table 4:** Evaluation of the pre-processing steps on the PadChest data set.

	**ResNet152**		**DenseNet201**		**Xception**		**InceptionV3**	
**Pre-processing: Cropping**	**G-Mean**	**AUC**	**G-Mean**	**AUC**	**G-Mean**	**AUC**	**G-Mean**	**AUC**
No	88.32%	96.03%	89.69%	96.73%	87.84%	95.30%	87.68%	95.47%
Yes	88.47%	96.05%	90.68%	96.94%	87.90%	95.62%	88.42%	95.86%

The numbers in bold depict the best overall performance across all evaluated architectures in terms of geometric mean (G-Mean) and area under the receiver operating characteristic curve (AUC).

During the unsupervised domain adaptation experiments, the described data sets (see Section 4.1) are used either as the source data set (S) or as the target data set (T). When used as the source data set, the training set (with its corresponding labels) specific to the data set is used throughout the optimization process. However, when used as target data set, the training set of the corresponding data set is used throughout the optimization process, without its labels in order to simulate an unlabeled set of data. The optimized and adapted model is subsequently evaluated on the testing set of the target data set.

In order to assess the performance of the proposed unsupervised domain adaptation approaches, further experiments are performed by optimizing a model on the source data set and applying the optimized model on the target data set, without any form of domain adaptation (S→T). An additional baseline is computed, by optimizing a model on the target data set and evaluating the model on the same target data set. In this scenario, the model is optimized using the training set of the target data set (this time with its corresponding labels), and subsequently evaluated on the corresponding testing set of the target data set (T→T). The results of the performed unsupervised domain adaptation experiments are depicted in Table 5.

**Table 5:** Classification performance.

	**ResNet152**			**DenseNet201**			**Xception**			**InceptionV3**		
	**Sens**	**Spec**	**G-Mean**	**Sens**	**Spec**	**G-Mean**	**Sens**	**Spec**	**G-Mean**	**Sens**	**Spec**	**G-Mean**
S**: CXR PadChest -** T**: CXR Ulm**												
S→T	95.00%	15.38%	38.23%	100%	15.38%	39.22%	100%	15.38%	39.22%	100%	15.38%	39.22%
DRDA	80.00%	46.15%	60.76%	90.00%	38.46%	58.83%	80.00%	46.15%	60.76%	95.00%	53.85%	71.52%
RDRDA	80.00%	46.15%	60.76%	90.00%	53.85%	69.62%	80.00%	46.15%	60.76%	95.00%	38.46%	60.45%
S**: CXR OpenI -** T**: CXR Ulm**												
S→T	95.00%	07.69%	27.03%	90.00%	23.08%	45.57%	90.00%	30.77%	52.62%	90.00%	30.77%	52.62%
DRDA	75.00%	61.54%	67.94%	75.00%	61.54%	67.94%	70.00%	69.23%	69.61%	85.00%	61.54%	72.32%
RDRDA	80.00%	61.54%	70.17%	60.00%	69.23%	64.45%	75.00%	61.54%	67.94%	85.00%	61.54%	72.32%
T→T	55.00%	76.92%	65.04%	60.00%	69.23%	64.45%	70.00%	69.23%	69.61%	65.00%	69.23%	67.08%
S**: CXR PadChest -** T**: CXR OpenI**												
S→T	85.71%	88.54%	87.12%	82.14%	90.83%	86.38%	86.90%	81.95%	84.39%	90.48%	88.54%	89.50%
DRDA	86.90%	87.97%	87.43%	89.29%	89.53%	88.77%	86.90%	80.52%	83.65%	91.67%	84.53%	88.02%
RDRDA	89.29%	87.11%	88.19%	85.71%	87.97%	86.83%	86.90%	81.95%	84.39%	92.86%	85.10%	88.90%
T→T	75.00%	91.12%	82.67%	71.43%	93.70%	81.81%	78.57%	87.97%	83.14%	83.33%	89.40%	86.31%

The numbers in bold depict the best overall performance across all evaluated architectures in terms of geometric mean (G-Mean), for each of the pre-trained models.

**Figure 4:**
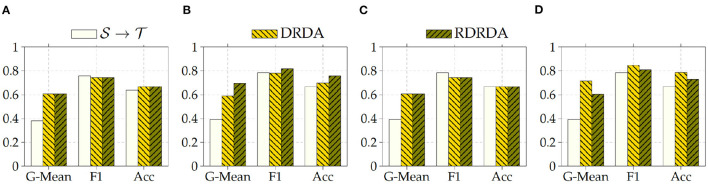
S: CXR PadChest-T: CXR Ulm. **(A)** ResNet152. **(B)** DenseNet201. **(C)** Xception. **(D)** InceptionV3.

**Figure 5:**
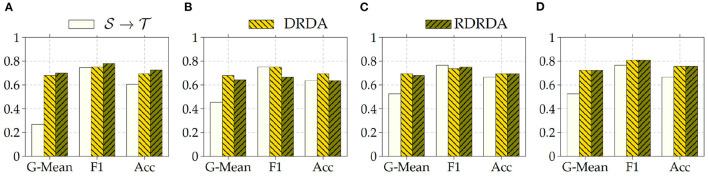
S: CXR OpenI-T: CXR Ulm. **(A)** ResNet152. **(B)** DenseNet201. **(C)** Xception. **(D)** InceptionV3.

Concerning the experiments conducted with the Ulm data set as target domain (T), it can be observed that uniquely optimizing a model on the source domain (either the PadChest data set or the OpenI data set) and applying the model directly on the target domain without any form of domain adaptation (S→T) results in very poor classification performances (which is an observable evidence of the domain shift). The model is biased into classifying almost every sample as belonging to the positive class with a Sensitivity rate attaining for some models a value of 100% while the Specificity rate is below 16%. However, applying either unsupervised domain adaptation approaches substantially improves the overall classification performance (see Figures 4, 5 for some better visualization).

Moreover, the depicted results show that the extent of the overall performance improvement depends on the pre-trained model used as backbone for the encoder (E). The best G-Mean performances could be achieved in both cases (PadChest and OpenI as source domain (S), respectively) by using a pre-trained InceptionV3 model as backbone. In the case of the PadChest data set as source domain, a Sensitivity score of 95.00% and a Specificity score of 53.85% could be achieved, with an overall G-Mean score of 71.52%. In the case of the OpenI dataset, a Sensitivity score of 85.00% and a Specificity score of 61.54% could be achieved, with an overall G-Mean score of 72.32%. Also, the performance of both domain adaptation approaches (DRDA and RDRDA) are very similar throughout the conducted experiments, even though DRDA outperforms RDRDA in most cases. Moreover, in both Figures 4, 5, it can be seen that the overall classification accuracy is concurrently improved with the G-Mean in most cases by applying either domain adaptation approaches. Meanwhile, models optimized on the OpenI data set in combination with domain adaptation, not just outperform those trained uniquely on the Ulm target domain (T→T), but also those trained while using the PadChest data set as source domain and evaluated on the Ulm target domain.

**Figure 6:**
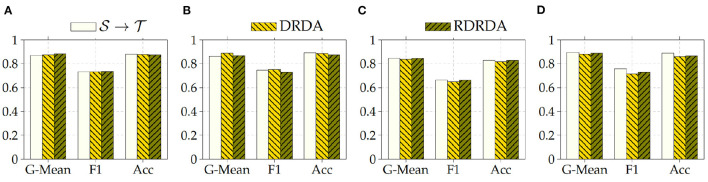
S: CXR PadChest-T: CXR OpenI. **(A)** ResNet152. **(B)** DenseNet201. **(C)** Xception. **(D)** InceptionV3.

Regarding the experiments conducted with the OpenI data set as target domain, similar results can be observed. In most cases, either of the domain adaptation approaches improves the classification performance in terms of G-Mean (with similar performances across all the evaluated models as can be seen in Figure 6), with the exception of experiments performed using a pre-trained InceptionV3 model as backbone. Across all performed experiments, RDRDA outperformed DRDA in most cases (in contrast to the previous observation regarding the Ulm data set). Still, the best overall performance could be achieved by using the InceptionV3 pre-trained model as backbone for the encoder (with a G-Mean of 89.50% without any form of domain adaptation and 88.90% with the RDRDA approach).

**Figure 7:**
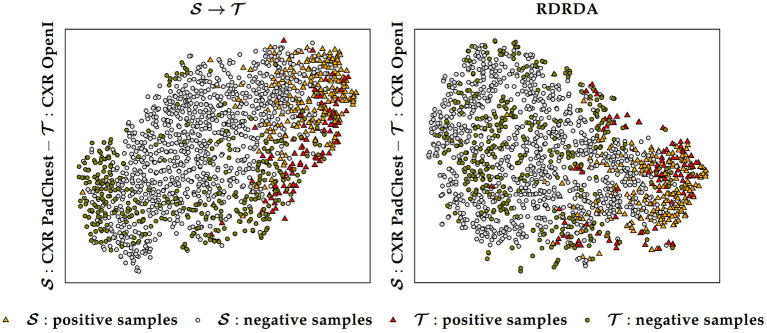
Source (S) and target (T) data distribution using a source only model (S→T) and a domain adaptation model (RDRDA), respectively. The features stemming from the first fully connected layer of the classification model are projected into a two-dimensional space using the t-SNE visualization approach. In this specific case the source domain consists of the PadChest data set and the target domain consists of the OpenI data set.

Additionally, it can also be observed that training the inference model on the PadChest data set without domain adaptation (S→T) results in a substantial performance improvement in comparison to the optimization of the model performed uniquely on the OpenI target domain (T→T) (in contrast to the Ulm data set where the same type of experiments resulted into poor performances, thus showing the extent of the dissimilarity between the data distribution of the Ulm data set and the data distribution of either the PadChest or OpenI data set). This is an indication that the PadChest data set is very similar to the OpenI data set with regards to the respective data distributions. This is further supported by the depiction of the data distribution of both PadChest and OpenI data sets in a two-dimensional space [generated by applying the t-distributed Stochastic Neighbor Embedding (t-SNE) (van der Maaten and Hinton, 2008) visualization approach] in Figure 7. The representation in this case is generated based on the feature representation stemming from the first fully connected layer of a classification model trained either without domain adaptation (S→T) or with domain adaptation (RDRDA). Each model is optimized by using the PadChest data set as the source domain and the OpenI data set as the target domain. Once the models are optimized, the feature representations of the respective testing sets are extracted and visualized using the t-SNE approach. In both cases, it can be observed that the generated feature representations are rather effective since it is clearly visible that both negative and positive samples are grouped into two slightly overlapping clusters. This is true for the source only model as well as the adapted model, hence the observed similarity of classification performances. The feature representations of the samples stemming from either source or target domains are also characterized by a similar overall sample distribution (see Figure 7), thus pointing at the similarity of both data sets.

## 6. Discussion

**Figure 8:**
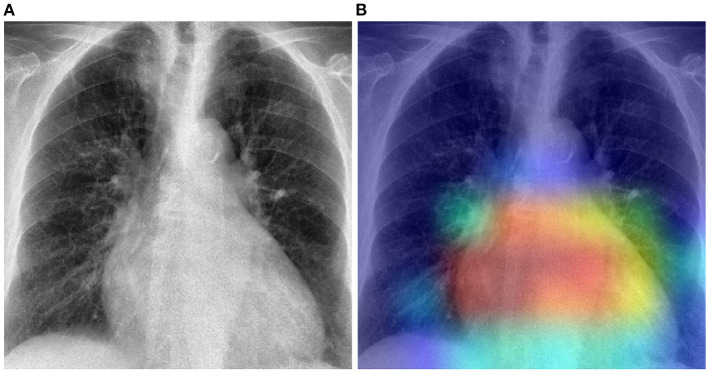
Gradient-weighted class activation mapping based on the RDRDA approach (with the InceptionV3 model as backbone). In this case, the OpenI data set was used as source domain and the Ulm data set as target domain: **(A)** Original CXR (Cardiomegaly). **(B)** Superimposed CXR with the corresponding class activation map.

The previously described results show that in a cross-domain setting, the domain shift negatively affects the overall performance of a classification model, particularly in cases where there is a huge dissimilarity between the data distributions of both the target domain and the source domain. In such cases, applying a model previously optimized on the source domain uniquely, without any form of domain adaptation, would result in very poor classification performances on the target domain. The proposed unsupervised domain adaptation approaches can effectively generate domain invariant feature representations, that can be subsequently and effectively used in both target and source domains to perform the underlying classification task. The results of the performed experiments have shown that the overall performance of the classification task can be substantially improved by applying either domain adaptation approaches. Moreover, in most cases, the unsupervised domain adaptation approaches also outperform models that are trained on a labeled set stemming from the target domain. Normally the baseline results (T→T) are considered as an upper bound for the domain adaptation results. However, in the current work, the amount of samples of the target domain is very small (in particular in the case of the Ulm data set). Hence, optimizing a deep neural network on such a small set remains challenging, even with transfer learning approaches. However, both the PadChest and the OpenI data sets have a significantly higher amount of labeled samples. Thus, the corresponding adapted models perform better than a model trained on labeled samples of the Ulm data set. This observation shows that knowledge can be transferred and successfully adapted from a source domain (with a large number of labeled samples) to a specific target domain with little to none labeled samples. This is particularly interesting in the area of medical image analysis, since most of the time, there is a huge amount of unlabeled data available, while the annotation process is known to be time- and resource-consuming. Such approaches can be used to adapt models trained on labeled data sets stemming from different centers and perform the labeling of a set of unlabeled samples. However, the overall performances of the presented approaches have to be significantly improved in order to enable a reliable annotation process. Furthermore, the overall performance of both unsupervised domain adaptation approaches are very similar. Overall, the RDRDA however slightly outperforms the DRDA approach. Since the regularization parameters (λ_*c*_, λ_*r*_, λ_*d*_ in Equation 2) were chosen empirically, it is believed that an improved and systematic optimization of these specific parameters can further improve the performance of the RDRDA approach. The depicted results also show that the overall performance of the classification task depends on the pre-trained model used as an encoder, with the InceptionV3 model outperforming the other assessed models in most cases. A depiction of the activation map corresponding to a case of cardiomegaly and generated by using an RDRDA approach (the InceptionV3 model is used as backbone in this case) with the OpenI data set as source domain and the Ulm data set as target domain, is presented in Figure 8. It can be observed that the output of the model is based on the heart-located region of the CXR.

## 7. Conclusion

As a summary, the proposed unsupervised domain adaptation approaches have proven to be effective in a cross-domain setting with domain shift, since a substantive performance improvement can be observed when adapting a model trained on the source domain to the target domain. In general, both approaches yield similar results. However, the experimental results show that the regularized deep reconstruction domain adaptation approach slightly outperforms its deep reconstruction domain adaptation counterpart. However, there is still some room for improvement and a thorough optimization of the regularization parameters of the loss functions is believed to be able to improve the overall performance of the RDRDA approach. Even if the proposed approaches are very simple and straight forward, other approaches involving generative adversarial networks such as Cycle-Consistent Adversarial Domain Adaptation (CyCADA Hoffman et al., 2018; CycleGAN Zhu et al., 2017) methods should also be assessed and compared with the proposed approaches, since they bring more flexibility to the domain adaptation process and have proven to be very effective in other image processing areas.

## Data availability statement

Publicly available datasets were analyzed in this study. This data can be found at: https://bimcv.cipf.es/bimcv-projects/padchest/; https://openi.nlm.nih.gov/gridquery?it=xg&coll=cxr&m=1&n=100.

## Author contributions

PT, LL, and HK designed the experiments. PT designed, implemented, and evaluated the unsupervised domain adaptation approaches and also collected and pre-processed the data used for the evaluation. CK, DB, AL, and MB collected, annotated, and assessed the data from the Ulm Medical Center. PT and HK wrote the first draft of the manuscript. MB and HK funding acquisition. All authors read and approved the manuscript.
